# Urinary exosome miR‐30c‐5p as a biomarker of clear cell renal cell carcinoma that inhibits progression by targeting HSPA5

**DOI:** 10.1111/jcmm.14553

**Published:** 2019-07-24

**Authors:** Shangqing Song, Manmei Long, Guopeng Yu, Yajun Cheng, Qing Yang, Jiayi Liu, Yiwei Wang, Jiayan Sheng, Linhui Wang, Zhong Wang, Bin Xu

**Affiliations:** ^1^ Department of Urology, Shanghai Ninth People's Hospital Shanghai Jiaotong University School of Medicine Shanghai China; ^2^ Department of Pathology, Shanghai Ninth People's Hospital Shanghai Jiaotong University School of Medicine Shanghai China; ^3^ Department of Urology, Changhai Hospital Second Military Medical University Shanghai China; ^4^ Department of Urology, Changzheng Hospital Second Military Medical University Shanghai China

**Keywords:** clear cell renal cell carcinoma, exosome, HSPA5, miR‐30c‐5p, urine

## Abstract

Exosome‐derived miRNAs are regarded as biomarkers for the diagnosis and prognosis of many human cancers. However, its function in clear cell renal cell carcinoma (ccRCC) remains unclear. In this study, differentially expressed miRNAs from urinal exosomes were identified using next‐generation sequencing (NGS) and verified using urine samples of ccRCC patients and healthy donors. Then, the exosomes were analysed in early‐stage ccRCC patients, healthy individuals and patients suffering from other urinary system cancers. Thereafter, the target gene of the miRNA was detected. Its biological function was investigated in vitro and in vivo*.* The results showed that miR‐30c‐5p could be amplified in a stable manner. Its expression pattern was significantly different only between ccRCC patients and healthy control individuals, but not compared with that of other urinary system cancers, which indicated its specificity for ccRCC. Additionally, the overexpression of miR‐30c‐5p inhibited ccRCC progression in vitro and in vivo. Heat‐shock protein 5 (HSPA5) was found to be a direct target gene of miR‐30c‐5p. The depletion of HSPA5 caused by miR‐30c‐5p inhibition reversed the promoting effect of ccRCC growth. In conclusion, urinary exosomal miR‐30c‐5p acts as a potential diagnostic biomarker of early‐stage ccRCC and may be able to modulate the expression of HSPA5, which is correlated with the progression of ccRCC.

## 1 INTRODUCTION

Renal cell carcinoma (RCC) is derived from renal epithelial cells. Clear cell renal cell carcinoma (ccRCC) is the most common subtype of RCC, which accounts for 86.95% of all cases in the population of China.^1^ Due to the poor benefits from surgical and systemic therapy for advanced kidney cancer,^2^, ^3^ it is essential to identify a biomarker that can be used to recognize early‐stage renal cancer.

MicroRNAs (miRNAs) are a class of small, endogenous RNAs of 21‐25 nucleotides in length. miRNA genes are mainly located in cancer‐associated genomic regions, which implies that some overexpressed miRNAs might be involved in promoting cancer progression.^4^, ^5^, ^6^, ^7^, ^8^ It has been demonstrated that miRNAs are significantly dysregulated in RCC; thus, it is reasonable to regard specific dysregulated miRNAs as potential biomarkers of RCC.^9^, ^10^, ^11^, ^12^

Exosomes are membrane‐bound vesicles with a diameter of 30‐140 nm that are secreted by almost all mammalian cell types and are present in body fluids such as blood, emulsion, spit and urine.^13^, ^14^ Various biological macromolecules such as lipids, proteins, mRNAs and miRNAs are included in exosomes.^13^, ^15^, ^16^ Therefore, exosomes that are secreted from tumour cells that contain cancer biomarkers can be used for early cancer diagnosis.^17^, ^18^, ^19^

In this study, we first detected the expression of dysregulated miRNAs in urine exosomes of ccRCC patients and healthy individuals, in order to identify a dysregulated miRNA that is specific to ccRCC. Furthermore, the biological function of this specific miRNA in the development and progression of ccRCC was investigated. In addition, the target gene of this miRNA was identified in order to explore its mechanism of action in ccRCC progression. This study is expected to provide a novel biomarker of early‐stage ccRCC, a theoretical basis for further pathological studies and the development of new therapeutic approaches for ccRCC.

## 2 METHODS AND MATERIALS

### 2.1 Patients and samples

Urine samples of 70 early‐stage (T1aN0M0) ccRCC patients, 30 early‐stage prostate cancer (T1N0M0) patients and 30 early‐stage bladder cancer (T1N0M0) patients prior to surgery at Shanghai Changzheng Hospital, Shanghai Changhai Hospital and Shanghai Ninth Peoples’ Hospital, ccRCC tissues and adjacent normal kidney tissues from 42 early‐stage ccRCC patients and urine samples from 30 healthy donors were collected. The clinical characteristics of the patients are shown in Table 1. Pathological data were confirmed by pathologists after operation. Patients who received chemotherapy or radiotherapy before sample collection were excluded. This study was approved by the Ethics Committees of Shanghai Jiao Tong University. Informed consent was obtained from all participants before the study. Additionally, all animals used were housed and treated under approved protocols.

**Table 1:** Clinical characteristics of patients and healthy people

	ccRCC	PCa	BCa	Healthy
Total number	70	30	30	30
Gender				
Female	35	0	15	15
Male	35	30	15	15
Age				
Median	55	58	52	51
Range	45‐68	52‐71	27‐63	48‐61
Stage				
Ⅰ	5	3	4	
ⅡA	51	21	18	
ⅡB	15	6	8	
TNM stage				
T1	45	13	8	
T2	25	17	22	
T3				
T4				
N				
N0	70	30	30	
N1‐3				
M				
M0‐M1	0	0	0	

Abbreviations: BCa, bladder cancer; ccRCC, clear cell renal cell carcinoma; PCa: prostate cancer.

### 2.2 Exosome isolation from human urine samples

Morning urine was collected and centrifuged (2000× *g* for 5 minutes) at 4°C, and then filtrated at 0.22 μm before being stored at −80°C. The exosomes were then isolated from the human urine samples. First, 50 mL of cell‐free urine samples was thawed on ice. Thereafter, the samples were ultracentrifuged at 150 000 × g, overnight at 4°C. Next, the exosome pellets were washed in 11 mL of phosphate buffer saline (PBS), followed by a second step of ultracentrifugation at 150 000× *g*, at 4°C for 2 hours. The supernatant was discarded, and the pelleted exosomes were then re‐suspended in 500 μL of TRIzol for RNA analysis or in 250 μL of lysis buffer (8 mol/L urea, 2.5% SDS, 5 mg/mL leupeptin, 1 mg/mL pepstatin and 1 mmol/L phenylmethylsulphonyl fluoride) for protein analysis. The exosomes used for transmission electron microscopy (TEM) were re‐suspended in 100 mL of PBS. 10 mL of this exosome sample was used for NanoSight LM10 (NanoSight Ltd.) analysis after Nano dilution 1:100 in PBS.

### 2.3 RNA isolation from cells and exosomes

RNA was isolated using a TRIzol Plus RNA Purification Kit (Life Technologies, 12183555) according to the manufacturer's protocol. After verifying the quality of total RNA using an Agilent 2100 Bioanalyzer RNA 6000 Pico Kit (Agilent Technologies), we proceeded to the second part of the protocol, small RNA enrichment from total RNA by increasing the ethanol content of the sample, followed by isolation over a glass‐fibre filter and elution. Thereafter, the quality of the small RNA samples was analysed using an Agilent 2100 Bioanalyzer Small RNA Kit (Agilent Technologies). RNA total concentration was measured using NanoDrop, and miRNA concentration was analysed using the Quant‐iT RiboGreen Kit (Thermo Scientific).

### 2.4 Next‐generation sequencing

Total RNA was isolated from exosomes using TRIzol LS, according to the manufacturer's instructions, and small RNA concentration was determined using a small RNA Bioanalyzer Chip (Agilent). Preparation and sequencing of the cDNA libraries was performed with 200‐600 ng of small RNA from the total RNA samples, according to the manufacturer's instructions (Illumina). The cDNA sequence library yield was measured using an Agilent 2100 Bioanalyzer (Agilent Technologies), and the samples were pooled in equimolar concentrations for the sequencing run. For sequencing, paired‐end 100 (PE100) cycles were performed on a HiSeq 2000 (Illumina).

### 2.5 Cell lines and cultures

The 786‐0, ACHN human renal cancer cell lines and human renal epithelial tubular cell line HK‐2 were purchased from the Institute of Cell Research, Chinese Academy of Sciences (Shanghai, China). The cells were cultured in RPMI 1640 medium (786‐0 and HK‐2) or DMEM (ACHN) supplemented with 10% (v/v) exosome‐depleted FBS (Gibco, Thermo Fisher Scientific) and 100 U/mL penicillin and 100 mg/mL streptomycin. All cell lines were kept in a humid atmosphere, with 5% CO_2_ and at 37°C.

### 2.6 Isolation of exosomes

Exosomes were obtained from the supernatant of cells, as previously described with a few modifications. In brief, cells were grown in 25‐cm^2^ flasks in exosome‐depleted FBS RPMI 1640 medium until they reached a confluency of 80%‐90%. Next, the medium was collected and centrifuged at 800× *g* for 5 minutes, followed by a centrifugation step of 2000× *g* for 10 minutes, in order to discard cellular debris. Thereafter, the medium was filtered using a 0.2 mm pore filter (syringe filter, 6786‐1302; GE Healthcare) and ultracentrifuged at 100 000× *g* for 2 hours at 4°C. The exosome pellets were washed with 5 mL PBS, followed by a second step of ultracentrifugation at 100 000× *g* for 2 hours at 4°C. The supernatant was then discarded. The exosomes that were to be used for RNA extraction were re‐suspended in 500 μL of TRIzol, while the exosomes that were to be used for protein extraction were re‐suspended in 250 μL of a lysis buffer. The exosomes that were to be used for TEM were re‐suspended in PBS. 10 mL of this exosomes sample was used for NanoSight LM10 (NanoSight Ltd.) analysis after dilution 1:100 in PBS.

### 2.7 Transfection of microRNA mimics, inhibitors, shHSPA5 and HSPA5 in vitro

The cells were seeded at a density of 2 × 10^5^ or 5 × 10^5^ per well. miR‐30c AgomiR (MiScript miRNA Mimics; Qiagen), AntagomiR (MiScript miRNA Inhibitor) and shHSPA5 were transfected using the Attractene transfection reagent at a concentration of 20 µmol/L by fast‐forward transfection, according to the manufacturer's recommendation (Qiagen). Transfection was carried out for 24 hours, followed by infection or co‐culture with infected cells. Prior to infection or co‐culture, the efficiency of transfection and cell viability was determined using real‐time PCR and MTS assay (Promega), respectively.

### 2.8 Protein mass spectrometry (MS) assay

Mass spectrometry assay included sample preparation (ACHN, 786‐O and HK‐2 cells), chromatography‐mass spectrometry and data processing. The Orbitrap Fusion Mass Spectrometer (Thermo Scientific) was used according to the manufacturer's instruction. More details were illustrated in the supplement.

### 2.9 Luciferase reporter assay

Light switch luciferase assay reagents were obtained from Promega Corporation, USA. Cells overexpressing miR‐30c‐5p were transfected together with a luciferase reporter plasmid and the pRL‐TK vector expressing Renilla luciferase for 24 hours. Luciferase activity was measured using a dual reporter assay system according to the manufacturer's instructions. Renilla luciferase was used for normalization.

### 2.10 Cell proliferation assay

After transfection, the viability of the cells was determined using a CCK‐8 assay. In brief, ACHN cells were seeded into 96‐well plates, at a concentration of 1 × 10^4^ cells/mL. After incubation at 37°C and 5% CO_2_ for 24, 48 and 72 hours, 10 µL of CCK solution was added to each well, which was followed by incubation at 37°C for 4 hours. Absorbance of each well at 450 nm was detected using a microplate reader.

### 2.11 Colony formation assay

Colony formation assays were performed with standard protocol. In brief, the cells (500/well) were cultured in 6‐well plates for 2 weeks. The colonies were then counted and photographed using Quantity One software (Bio‐Rad).

### 2.12 Western blotting

Cells were lysed in a RIPA buffer containing 5 mg/mL leupeptin, 1 mg/mL pepstatin and 1 mmol/L phenylmethylsulphonyl fluoride. The exosomes were lysed in 8 mol/L urea, 2.5% SDS containing 5 mg/mL leupeptin, 1 mg/mL pepstatin and 1 mmol/L phenylmethylsulphonyl fluoride. The protein blot was blocked for 1 hour at room temperature with 5% non‐fat dry milk in PBS‐0.05% Tween, and incubated overnight at 4°C with 1:300 anti‐CD63 (sc‐166029, Santa‐Cruz) and 1:300 anti–β‐actin (A3854, Sigma‐Aldrich) primary antibodies. Thereafter, it was incubated for 1 hour at room temperature with horseradish peroxidase (HRP)‐conjugated secondary antibodies. After incubation with the antibodies, the protein blot was washed on an orbital shaker, four times at 10‐minute intervals, with PBS‐0.05% Tween‐20. The blots were developed with chemiluminescent reagents from Pierce.

### 2.13 Quantitative reverse transcription‐polymerase chain reaction

Urine exosome RNA (50 ng/sample) was used for reverse transcription, which was performed according to the manufacturer's instructions (Life Technologies). miRNA assay #4427975 from Life Technologies was used as the primer for the mature sequences. qRT‐PCR was performed according to the manufacturer's instructions.

### 2.14 Immunohistochemistry analysis

Formalin‐fixed paraffin‐embedded (FFPE) tissue was cut into 3‐μm sections for immunohistochemistry (IHC). Slides were deparaffinized with xylene and hydrated with decreasing concentrations of an ethanol solution. Sections were treated with citrate buffer solution (Maixin‐Bio) at 100°C for 1 minutes for antigen retrieval and were permeabilized in 3% hydrogen peroxide for 10 minutes at 37°C. Slides were then incubated with primary antibodies at 4°C overnight, followed by incubation with secondary antibodies for 60 minutes at 37°C. Finally, 3,3‐diaminobenzidine tetra‐hydrochloride was used as colouring reagent, and haematoxylin was used as a counterstain for nuclei. The stained fields were photographed using a light microscope equipped with a camera (Olympus). The primary antibody used for IHC was anti‐GRP78 (1:200, ab108613, Abcam). Staining intensity was quantified using ImageJ software (NIH).

### 2.15 Tumorigenicity assay

The tumour formation ability of the RCC cells overexpressing miR‐30c‐5p was evaluated by injecting cell suspensions into BALB/c nude male mice. The 10 mice were randomly divided into an overexpressing group and a control group. For each mouse, 2 × 10^7^ of ACHN cells (stably transfected with the miR‐30c‐5p mimics or control) were injected into the buttocks. After 4‐5 weeks, the mice were killed and the tumours were weighed.

### 2.16 Statistical analyses

All experiments were performed in triplicate. All data are presented as mean ± standard deviation. Comparison between groups was conducted using Student's *t* test or chi‐squared test. All statistical analyses were performed with SPSS software (SPSS 20, IBM). A *P* value <.05 was considered statistically significant.

## 3 RESULTS

### 3.1 Characterization of urinary exosomes and miRNA expression of urinary exosomes

**Figure 1:**
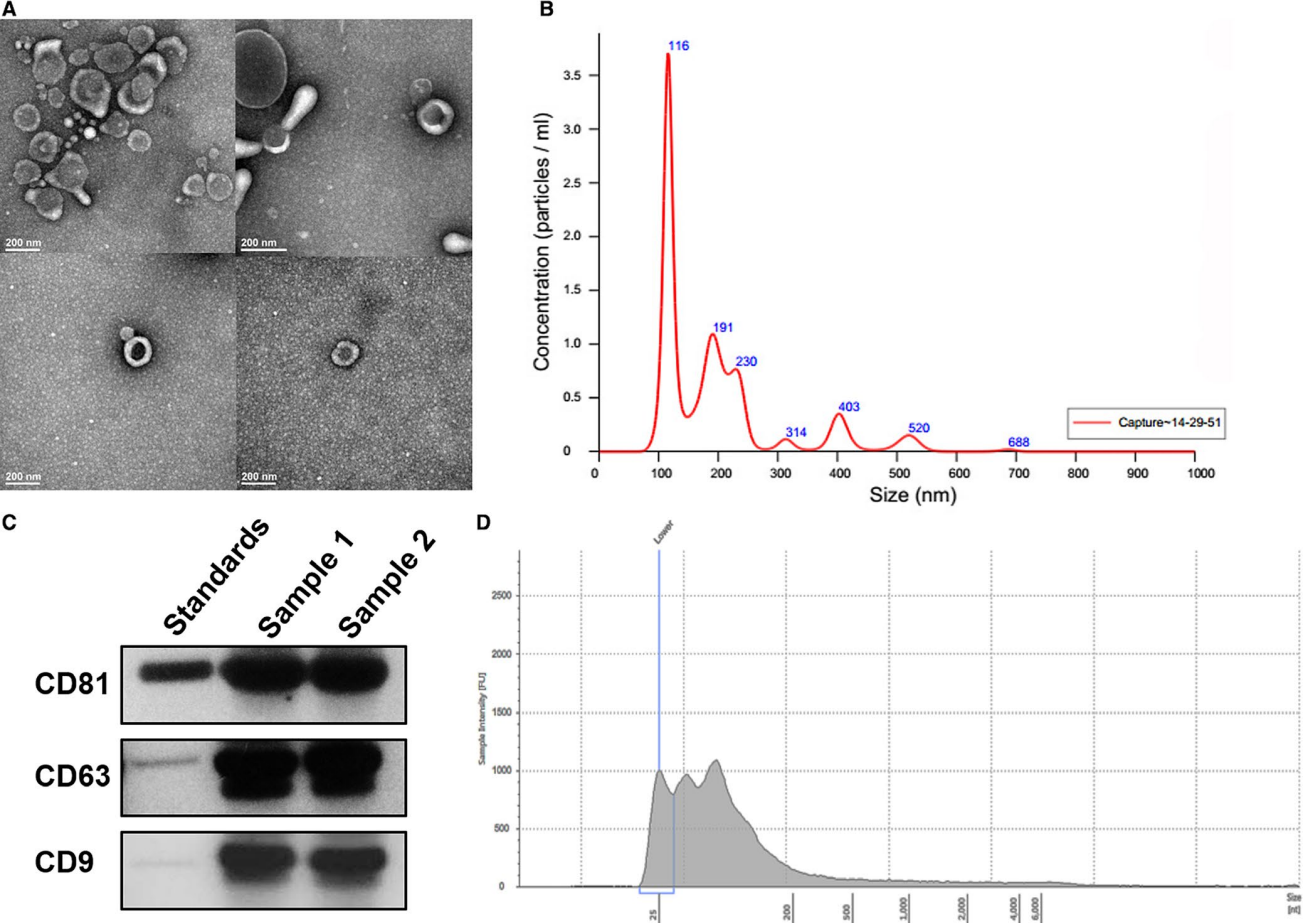
Identification and characterization of exosomes. A, Urinary exosomes were isolated from the urine collections using differential ultracentrifugation as described in Methods. Urinary vesicles, showing the characteristic exosomal cup shape and size, are shown in the electron micrograph; scale bar = 100 nm. B, The size and concentration of the urinary exosomes were determined using NTA. C, The expression of exosomal markers was assessed using immunoblotting of total protein extracts from isolated urinary exosomes. D, RNA profiles were obtained using an Agilent 2100 Bioanalyzer. All experiments were performed in triplicate

In order to detect the miRNA expression of urinary exosomes, exosomes were obtained from urine and were examined using electronic microscopy, and the characteristic cup‐shaped morphology of the vesicles was observed (Figure 1A). Nanosight tracking analysis (NTA) displayed that the diameters of most vesicles were around 100 nm (Figure 1B). Then, specific markers (CD9, CD81, CD63) of urine exosomes were detected in the urine samples using Western blotting (Figure 1C), which verified the exosomes and its purity. Furthermore, the RNA profile isolated from the urine exosomes was assessed using Bioanalyzer, and the results show that small RNAs are the main RNA fraction observed in the exosomes and that the ribosomal RNA peaks were undetectable (Figure 1D). These results confirm the integrity and purity of the miRNAs of the exosomes obtained through the isolating operation.

### 3.2 Identification of 16 potential miRNA biomarkers in urine exosomes of ccRCC patients

**Figure 2:**
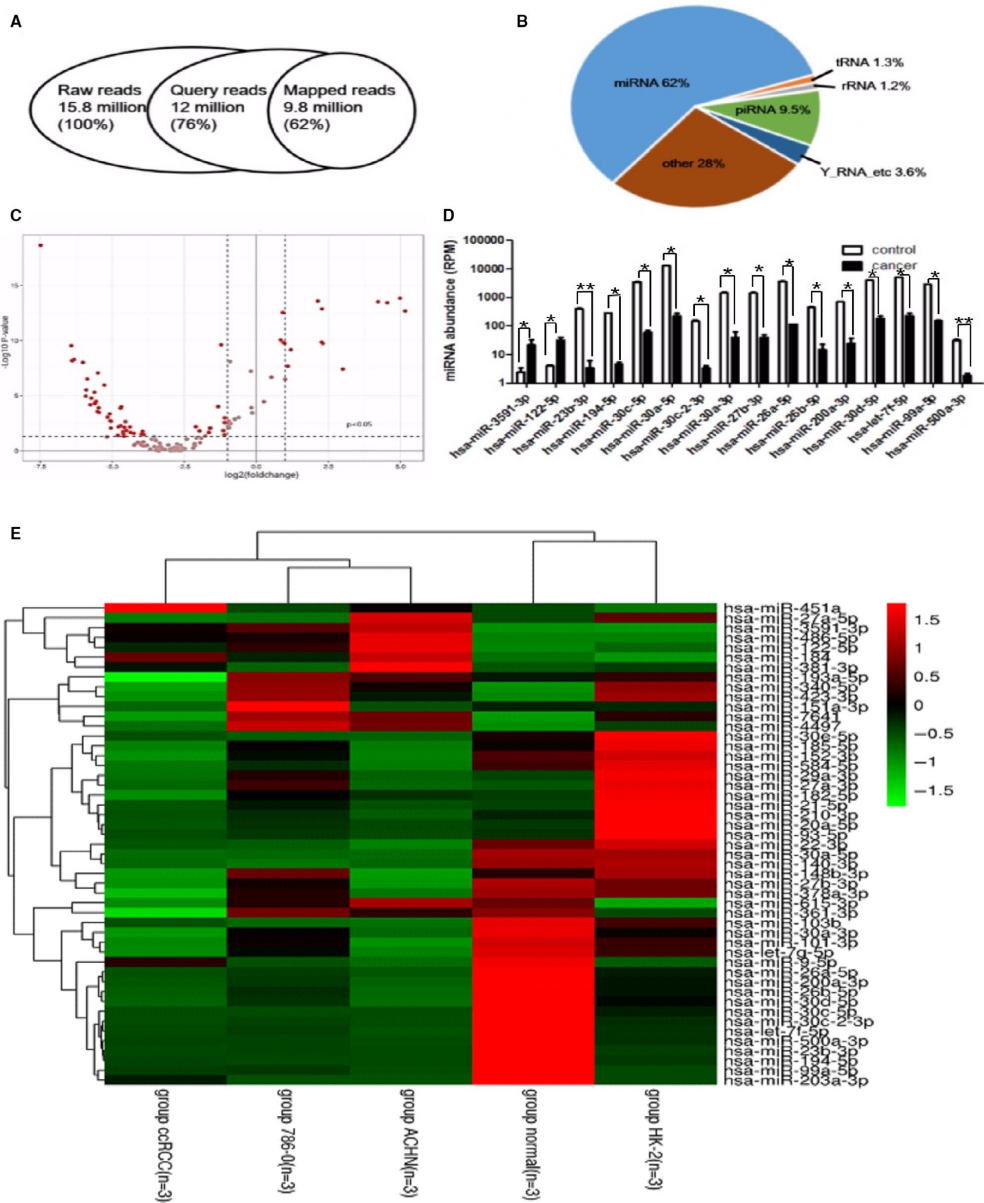
Exosome identification of RNA sequencing. A, Overview of deep sequencing results, including the number of reads, mapped sequencing reads and the distribution of mapped reads between different ncRNA classes. B, miRNA (62%), piRNA fragments (9.5%), tRNA fragments (1.3%), Y‐RNA fragments (3.6%), rRNA fragments (1.2%) and other fragments (28%). C, Volcano plot showing differences between urine exosome miRNAs of healthy individuals (n = 10) and RCC (n = 15) patients. The miRNAs were classified according to fold changes (log2 FC) between healthy individuals and RCC patients. Vertical dotted lines: miRNA with >2‐fold enrichment in urine exosomes of healthy individuals or RCC patients. D, Top 16 miRNAs that were significantly differentially expressed in healthy control individuals compared with those of cancer patients (*P < .05, **P < .01). E, Heatmap of the 49 miRNAs differentially expressed in exosomes from urine and cells. All experiments were performed in triplicate

In order to study the expression pattern of exosomal miRNA in the samples from ccRCC patients and healthy control individuals, total RNA was first isolated from the urine exosomes. The contents of the small RNAs in the exosomes of ccRCC patients and healthy control individuals were found to be consistent. Thereafter, miRNAs were isolated from total RNA and next‐generation sequencing (NGS) was performed, in order to compare the urinary exosome miRNA expression pattern of ccRCC patients with that of healthy individuals. The samples were then analysed using an ultrasequencer Illumina HiSeq 2000, a platform that can deliver high‐quality data. After quality controls and adapter elimination (Figure 2A), only high‐quality reads were aligned using a mirDeep2 algorithm against the mirBase v20 database, for the identification and quantification of previously described miRNAs (Figure 2B). In total, 126 miRNA species were identified (Table S1).

The total number of valid sequence reads was used as a measure of its relative abundance. Data were normalized by Z‐score to compare relative expression in urinary exosomes of ccRCC patients and normal persons, and the volcano plot (Figure 2C) shows the different preliminary expressions of the miRNAs. Among them, 16 miRNAs were found to have significantly different (*P* < .05) expressions between patients with cancer and healthy control individuals, as shown in Figure 2D, which were part of the candidate to screen the biomarker.

In order to verify the different expressed miRNAs were connected to the ccRCC, we detected the renal carcinoma cell lines 786‐0 and ACHN, and renal epithelial cell lines HK‐2. The exosomes were obtained from cultured cells, and miRNA was sequenced. We identified 297 known miRNA species and screened 116 of them which expressed significant diverse effects (*P* < .01).

Comparing the sequencing data of urinary exosomes and cell exosomes, 49 miRNAs were found to have similar expression trend (Table S2). They were down‐regulated or up‐regulated both in urinary exosomes and in cell exosomes, and the similar trend indicated the definite law of different expression. The heatmap of the 49 microRNA respective expression in exosomes of ccRCC patients and normal people, 786‐0, ACHN and HK‐2 cell lines is shown in Figure 2E. The 49 miRNAs were the other part of candidate, and the target biomarker miRNA would be screened from them.

### 3.3 miR‐30c‐5p may be a specific biomarker of RCC‐derived urinary exosomes

**Figure 3:**
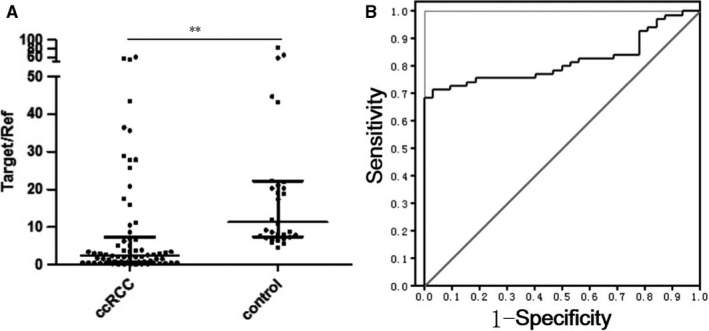
Clinical performance of renal carcinoma cell evaluation of urine exosome miR‐30c‐5p. A, RT‐PCR validation of the expression of miR‐30c‐5p, showing significant differences (*P* = .0012) between healthy control individuals and ccRCC patients. B, ROC curve based on urinary exosomal miR‐30c‐5p levels of ccRCC patients (n = 70) and healthy individuals (n = 30). All data are expressed as mean ± SD. **P < .01. All experiments were performed in triplicate

From the two groups of candidate, 16 miRNAs showed dramatically different expression in urinary exosome and 49 miRNAs showed similar different expression trend both in urinary and in cellular exosomes, in which 5 overlapped miRNAs, miR‐30c‐5p, miR‐27b‐3p, miR‐26a‐5p and miR‐194‐5p, which were down‐regulated, and miR‐122‐5p, which was up‐regulated, were screened. In order to validate the data obtained by NGS and find a potential biomarker of early‐stage RCC diagnosis, the five miRNAs mentioned above were tested using qRT‐PCR performed on urine and urine exosomes of 70 ccRCC patients and 30 healthy individuals. Among them, miR‐194‐5p and miR‐26a‐5p were barely detectable and the expression levels of miR‐30c‐5p, miR‐122‐5p and miR‐27b‐3p were stably amplified (data not shown). In order to identify a specific biomarker that can distinguish between ccRCC patients and patients with other urinary system cancers, miRNAs of the urine exosomes of 30 prostate cancer patients and 30 bladder cancer patients were then evaluated. Although miR‐30c‐5p (3′‐CGACUCUCACAUCCUACAAAUGU‐5′) was stably amplified in urine exosomes of prostate cancer and bladder cancer patients as well, there was no significant difference between its expression in these patients and healthy individuals. However, the expression level of miR‐30c‐5p in the urinary exosomes of ccRCC patients was significantly lower than that of normal individuals (Figure 3A), which implies that miR‐30c‐5p might be a potential urinary exosomal ccRCC biomarker. Furthermore, a receiver operating characteristic (ROC) curve revealed that the area under the curve (AUC) was 0.8192 (95% confidence interval was 0.7388‐0.8996, *P* < .01) (Figure 3B). The sensitivity and specificity of urinary exosome miR‐30c‐5p in the diagnosis of ccRCC were found to be 68.57% and 100%, respectively.

### 3.4 Attenuation of miR‐30c‐5p promotes the progression of ccRCC

**Figure 4:**
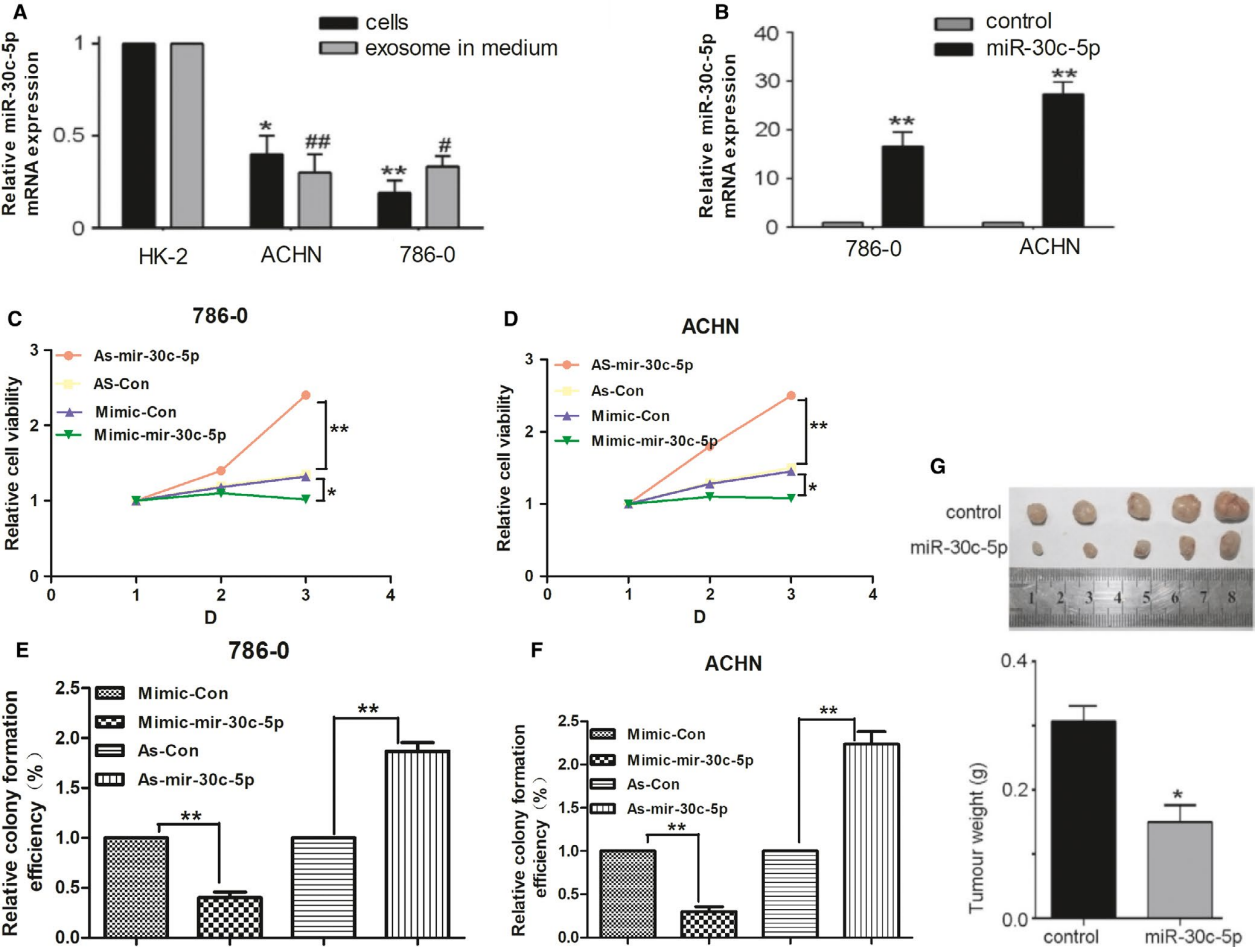
Biological behaviour evaluation of renal carcinoma cell miR‐30c‐5p. A, miR‐30c‐5p expression in renal carcinoma cells and medium was evaluated using qRT‐PCR. B, miR‐30c‐5p mimics were transfected with renal carcinoma cells (768‐O and ACHN cell lines), and transfection efficiency was measured using qRT‐PCR. (C‐D), 786‐O and ACHN cell lines were transfected with miR‐30c‐5p mimic, inhibitor or negative control, and cell proliferation was measured by CCK‐8 assays. (E‐F), Colony formation assay in RCC cells transfected with miR‐30c‐5p mimic, inhibitor or negative control. G, Tumorigenicity assay of nude mice was performed on the miR‐30c‐5p mimic group and the control. All data are expressed as mean ± SD. *P < .05, **P < .01, ^#^ *P *< .05 and ^##^ *P *< .01. All experiments were performed in triplicate

In order to study the role of miR‐30c‐5p in the progression of ccRCC, the expression of miR‐30c‐5p and exosomal miR‐30c‐5p was evaluated in RCC cell lines including 786‐O, ACHN and the human renal epithelial tubular cell line HK‐2, as well as their mediums. miR‐30c‐5p expression was consistently down‐regulated in 786‐O and ACHN cells compared with that of HK‐2, and exosomal miR‐30c‐5p was down‐regulated in the medium of RCC cells compared with that of the medium of the HK‐2 cells (Figure 4A). 786‐O and ACHN cells were then transfected with miR‐30c‐5p mimics, and transfection efficiency identified using qRT‐PCR showed that miR‐30c‐5p expression increased in cells transfected with miR‐30c‐5p mimics, compared with that of the control (Figure 4B). Cell proliferation assays showed that compared with the group transfected with the mimic control, 786‐O and ACHN cells transfected with miR‐30c‐5p mimic had greatly reduced cell viability and colony formation efficiency (Figure 4C‐F). A similar trend was also observed in vivo. miR‐30c‐5p overexpression inhibited the tumorigenicity of nude mice with ACHN xenografts (Figure 4G). The results above suggest that miR‐30c‐5p inhibits ccRCC progression both in vitro and in vivo.

### 3.5 miR‐30c‐5p directly targets HSPA5, and the progression of ccRCC inhibited by miR‐30c‐5p is regulated by HSPA5

In order to further clarify the potential mechanism of ccRCC progression inhibited by exosomal miRNA, the gene expression of the ccRCC cell lines and normal renal cell lines was evaluated at protein level using protein mass spectrometry analysis, as protein is one of the most common regulation targets of miRNAs. The MS analysis detected 1009 proteins and found about 110 differentially expressed proteins, of which the grouping is shown in Figure S1. Meanwhile, potential target genes of miR‐30c‐5p were scanned for using four online programs: miRanda (http://microrna.sanger.ac.uk/targets/v5/), miRDB (http://www.mirdb.org/miRDB/), TargetScan (http://www.targetscan.org/) and CLIP (http://starbase.sysu.edu.cn/). As a result, a total of 6409 target genes were screened out. 30 genes were found to be potential target genes, by comparing the protein MS with online predictions, for simultaneously showing differential expression through MS and being predicted by at least one online program. Among them, HSPA5 was predicted by two online programs (miRanda and CLIP) and showed an obviously different expression between the RCC cell line and the HK2 cell line (3.8915‐fold up‐regulated in 786‐O and 4.387‐fold up‐regulated in ACHN). In addition, HSPD1 was also predicted by two online programs (miRanda and CLIP) and showed a significantly higher expression in the RCC cell line (6.139‐fold up‐regulated in 786‐0 and 6.021‐fold up‐regulated in ACHN) (data not shown).

**Figure 5:**
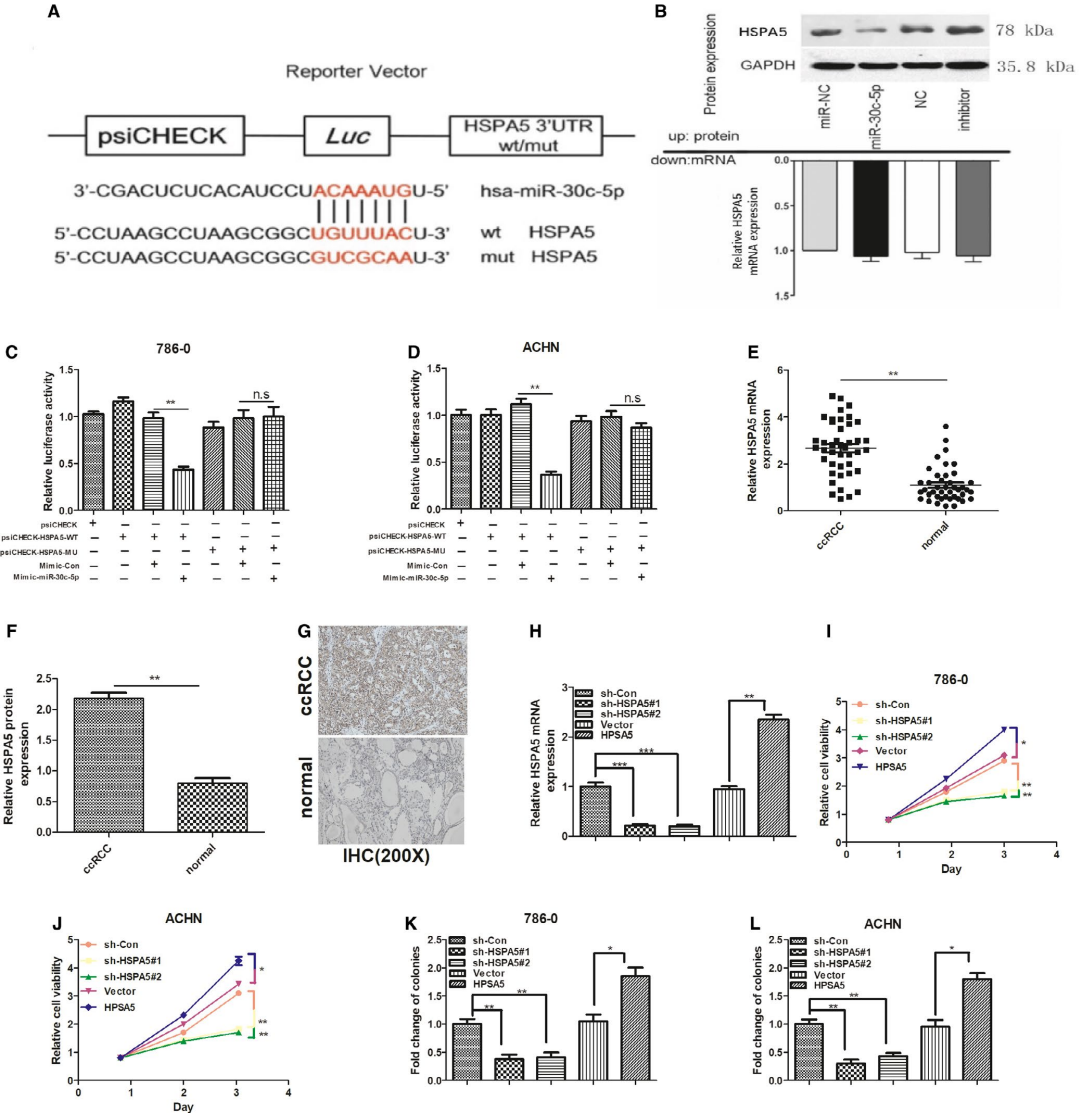
HSPA5 is a target gene of miR‐30c‐5p. A, Schematic representation of 3′‐UTR of HSPA5 mRNA reporter with and without the miR‐30c‐5p seed‐binding site (red). B, HSPA5 protein expression (up) and mRNA levels (down) of ACHN cell lines infected with miR‐30c‐5p were measured. (C‐D), The relative luciferase activities of either the WT or Mut‐3′‐UTR of the HSPA5 reporter in combination with the miR‐30c‐5p mimic in 786‐O and ACHN cells. (E‐F), qPCR and IHC analyses of the mRNA and protein levels of HSPA5 in 42 paired ccRCC and adjacent normal kidney tissues. G, Representative images of IHC staining in ccRCC and adjacent normal kidney tissues. H, qPCR analysis of the expression level of HSPA5 after knocking down it with shRNA and transfecting with HPSA5. (I‐J), 786‐O and ACHN cell lines were transfected with HSPA5 shRNAs, negative control, vector and HPSA5, and cell proliferation was measured by CCK‐8 assays. (K‐L), Colony formation assays of RCC cells transfected with HSPA5 shRNAs, negative control, vector and HPSA5. All data are expressed as mean ± SD. **P < .01 and ***P < .001. All experiments were performed in triplicate

In order to confirm whether HSPA5 or HSPD1 would be the direct target gene of miR‐30c‐5p, luciferase activity assay was performed with ccRCC cells, which were transfected with luciferase constructs containing WT‐3′‐UTR and Mut‐3′‐UTR of HSPA5 (Figure 5A). Overexpression of miR‐30c‐5p substantially reduced the expression of HSPA5, while the miR‐30c‐5p inhibitor increased the HSPA5 at protein level (Figure 5B, up). However, the qRT‐PCR analysis showed that neither miR‐30c‐5p overexpression nor miR‐30c‐5p inhibitor transfection had a significant effect on HSPA5 at mRNA level (Figure 5B, down), suggesting that miR‐30c‐5p specifically regulated HSPA5 expression at the post‐transcriptional level. Furthermore, dual‐luciferase assays showed that while miR‐30c‐5p suppressed the luciferase activity of the reporter containing wt‐3′‐UTRs of HSPA5 in both 786‐O and ACHN cells, the effect was obviously abrogated with the mutated reporter (Figure 5C,D), whereas there was no effect on HSPD1 wt‐3′‐UTRs (data not shown), indicating that miR‐30c‐5p suppressed the expression of HSPA5 by directly binding to target sites in 3′‐UTRs. These results support the bioinformatics predictions and demonstrate that miR‐30c‐5p directly targets HSPA5 and inhibits its expression in ccRCC. Numerous studies have reported that HSPA5 performs an important role in the progression of various cancers.^20^, ^21^, ^22^, ^23^ Thus, we speculate that HSPA5 might be a direct target of miR‐30c‐5p in regulating the progression of ccRCC. To examine the biological significance of HSPA5 in ccRCC, correlations between HSPA5 expression levels and the pathologic characteristics of ccRCC were analysed. Via qPCR and IHC assays, we found that both HSPA5 mRNA and protein levels were higher in ccRCC tissues than normal kidney tissues (Figure 5E‐G). The expression level of HSPA5 was obviously decreased after knocking down it with shRNA and increased significantly after transfecting with HSPA5 (Figure 5H). Subsequently, reduced HSPA5 expression levels led to decreased cell viability and colony formation ability in 786‐O and ACHN cells, while increased HSPA5 expression levels may enhance cell viability and colony formation ability in both cells (Figure 5I‐L). Taken together, these results suggest that HSPA5 is a functional target of the miR‐30c‐5p.

## 4 DISCUSSION

Various studies have focused on the association between miRNA and cancers, while increasing evidence has shown a close connection between the dysregulated expression of miRNAs and the occurrence and progression of cancers. miRNAs derived from exosomes show properties of tumour specificity and sensitivity, which has been attracting an increasing amount of attention from researchers.

In this study, 16 miRNAs that were dissimilarly expressed between ccRCC patients and healthy individuals were first identified in urine exosomes. Next, we found that urinary exosome miR‐30c‐5p was the only one that was not differentially expressed in other urinary system cancers, which indicates its specificity for ccRCC. Moreover, the expression level of miR‐30c‐5p in urinary exosomes of ccRCC patients was lower than that of normal individuals, and a ROC curve revealed that the AUC value was 0.8192 (95% confidence interval was 0.7388‐0.8996, *P* < .01). Thus, we propose miR‐30c‐5p to be a potential candidate for a ccRCC biomarker. To our knowledge, this is the first time the correlation between miR‐30c‐5p and kidney cancer has been studied. Although most studies have focused on tumour tissues, we screened miRNAs from urinary exosomes, as one of the merits of using miRNA from urinary exosomes is its non‐invasiveness, which could especially contribute to the diagnosis of early‐stage ccRCC.

From the result that miR‐30c‐5p in urinary exosome of ccRCC patients was lower than healthy individuals, we then speculated that miR‐30c‐5p might act as a suppressor in ccRCC progression. As expected, overexpression of miR‐30c‐5p was found to inhibit the growth of RCC cells in vitro, indicating its association with the development and progression of ccRCC. Moreover, miR‐30c‐5p overexpression inhibited tumorigenicity in nude mice. Protein MS and RNA online programs were then used to speculate the regulation target of miR‐30c‐5p, thereby further exploring the molecular mechanism of dysregulation of miR‐30c‐5p in ccRCC. Cao^24^ reported that miR‐30c‐5p suppressed tumorigenesis and metastasis via MTA1 (metastasis‐associated protein 1) in gastric cancer. However, in the present study, HSPA5, also called GRP78/BiP, was confirmed to be a direct target of miR‐30c‐5p, as HSPA5 down‐regulation caused by miR‐30c‐5p inhibition reversed the promoting effect of ccRCC growth.

Heat‐shock protein 5 is a central regulator of the unfolded protein response of the endoplasmic reticulum. A number of studies have demonstrated the function that HSPA5 plays in cancer metastasis and in anti‐apoptotic process of cancer cells.^25^ HSPA5 expression positively correlates with the large tumour size, aggressiveness, high clinical stage and resistance to conventional chemotherapy of RCC.^26^ Additionally, several studies have focused on the regulation mechanism or signalling pathway of the differential expression of HSPA5 in cancers. Chang et al found that when E1A was expressed in cancer cells, p300 was activated to reduce acetylated HSPA5 levels and enhance its binding to GP78, thereby promoting HSPA5 ubiquitination and the subsequent inhibition of metastasis.^27^ It was also proven by Chen et al that the up‐regulation of HSPA5 was critical for high‐risk metastasis of breast cancer, as well as triple‐negative breast cancer, while they also found that HSPA5 might be a crucial mediator of the E1A‐suppressed metastatic ability of breast cancer cells.^28^ Meanwhile, Luo et al reported that enhanced cell migration and invasion by FOXM1 was significantly attenuated by the depletion of HSPA5 in colorectal cancer cells and that FOXM1‐triggered colorectal cancer cell migration and invasion was involved in the activities of cell‐surface HSPA5. Consequently, FOXM1‐HSPA5 signalling may be considered as a molecular target for designing novel therapeutic regimes for controlling colorectal cancer metastasis and progression.^29^ Chang et al provided new mechanistic insights into the understanding that deacetylation of HSPA5 by HDAC6 facilitates GP78‐mediated HSPA5 ubiquitination and suggested that the post‐translational regulation of the HSPA5 protein is critical for HSPA5‐mediated metastatic properties of breast cancer.^30^ Taken together with the results of our present study, it is not a surprise that HSPA5 also plays an important role in ccRCC progression and appears to be an interesting alternative target in cancer treatment. In conclusion, miR‐30c‐5p‐HSPA5 signalling pathway may be correlated with the progression of ccRCC, which indicates that urinary exosomal miR‐30c‐5p may act as a specific and sensitive biomarker for diagnosing and monitoring the progression of ccRCC.

## CONFLICT OF INTERESTS

The authors have no conflict of interest to disclose.

## AUTHOR CONTRIBUTIONS

Wang Zhong and Xu Bin designed the research; Song Shangqing, Long Manmei, Cheng Yajun and Yu Guopeng performed the experiments; Wang Linhui, Yang Qing, Liu Jiayi and Wang Yiwei contributed new reagents or sample collection; Song Shangqing, Cheng yajun and Shen Jiayan analysed the data and prepared figures; Song Shangqing, Xu Bin and Wang Zhong drafted the manuscript; all authors reviewed the manuscript.

## Data Availability

The data used to support the findings of this study are available from the corresponding author upon request.
