# Supplementary figures and images for: Urinary exosome miR‐30c‐5p as a biomarker of clear cell renal cell carcinoma that inhibits progression by targeting HSPA5
Source: J Cell Mol Med. 2019 Jul 24;23(10):6755–65. doi: 10.1111/jcmm.14553 (PMC6787446; doi:10.1111/jcmm.14553)

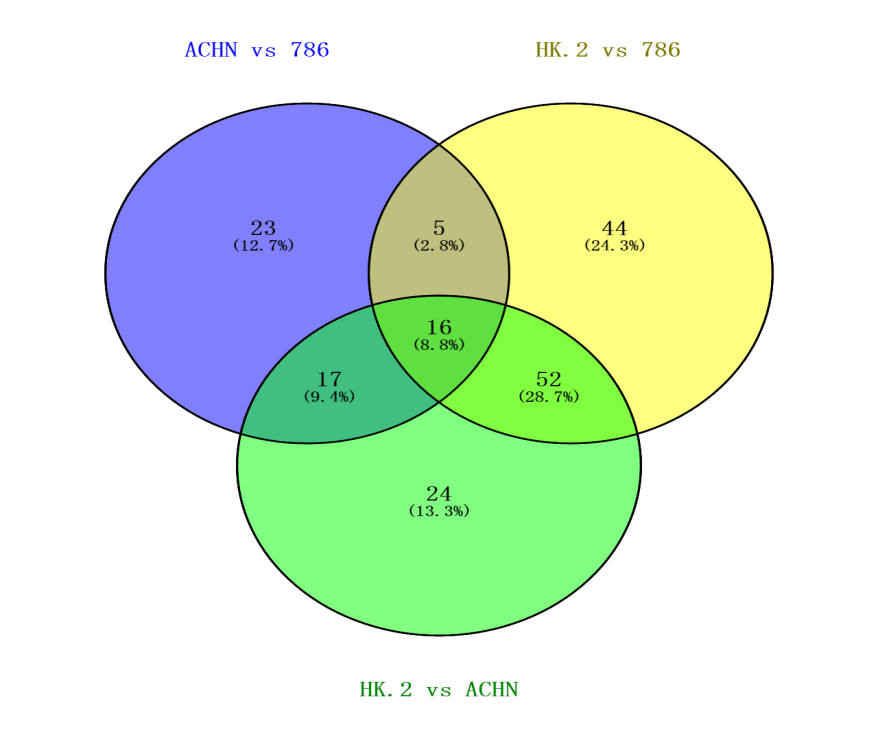

Supplement: Supplementary file 1 [file JCMM-23-6755-s001.tif]
