# Supplementary material for: Urinary exosome miR‐30c‐5p as a biomarker of clear cell renal cell carcinoma that inhibits progression by targeting HSPA5
Source: J Cell Mol Med. 2019 Jul 24;23(10):6755–65. doi: 10.1111/jcmm.14553 (PMC6787446; doi:10.1111/jcmm.14553)
Supplement: Supplementary file 2 [file JCMM-23-6755-s002.docx]

**Table S1 The miRNAs identified in urinary exosome in NGS**

| miRNA (ID) | log2 (fold change) | P-value | miRNA (ID) | log2 (fold change) | P-value |
| --- | --- | --- | --- | --- | --- |
| hsa-miR-23b-3p | -7.4856 | 18.6445649 | hsa-miR-615-3p | -1.6028 | 2.068494914 |
| hsa-miR-4488 | 4.9855 | 13.83711468 | hsa-let-7g-5p | -4.5111 | 1.843403455 |
| hsa-miR-3591-3p | 2.1315 | 13.57097887 | hsa-miR-1307-3p | -1.1454 | 1.842465817 |
| hsa-miR-143-3p | 4.217 | 13.51616225 | hsa-miR-224-5p | -1.9696 | 1.815080475 |
| hsa-miR-619-5p | 4.5396 | 13.44480763 | hsa-let-7d-5p | -3.9481 | 1.770907454 |
| hsa-miR-122-5p | 2.2755 | 12.87208326 | hsa-miR-152-3p | -4.627 | 1.720387506 |
| hsa-miR-4532 | 5.1595 | 12.68327059 | hsa-miR-28-3p | -4.3689 | 1.665693408 |
| hsa-miR-126-3p | 0.9172 | 12.53350176 | hsa-miR-340-5p | -1.651 | 1.664061131 |
| hsa-miR-486-5p | 0.8428 | 10.05802663 | hsa-miR-191-5p | -4.2513 | 1.577189387 |
| hsa-miR-4497 | 2.2637 | 9.856378124 | hsa-miR-101-3p | -4.6186 | 1.554342434 |
| hsa-miR-1290 | 0.9483 | 9.815311591 | hsa-miR-378a-3p | -3.9957 | 1.511213475 |
| hsa-miR-451a | 0.9835 | 9.742205388 | hsa-miR-140-3p | -3.9119 | 1.475575964 |
| hsa-miR-184 | 2.3063 | 9.736605783 | hsa-miR-514a-3p | -1.1059 | 1.453609756 |
| hsa-miR-9-5p | -1.2244 | 9.613777245 | hsa-miR-99a-5p | -4.8102 | 1.410742042 |
| hsa-miR-194-5p | -6.4189 | 9.530388618 | hsa-miR-193a-5p | -1.7733 | 1.34546139 |
| hsa-miR-127-3p | 1.1968 | 9.163810045 | hsa-miR-10b-5p | -4.5895 | 1.325141056 |
| hsa-miR-30a-5p | -6.3239 | 8.283838737 | hsa-miR-29a-3p | -3.5478 | 1.266058988 |
| hsa-miR-30c-5p | -6.3907 | 8.181800798 | hsa-let-7c-5p | -5.1843 | 1.247579017 |
| hsa-miR-9-3p | -0.9134 | 8.099101807 | hsa-let-7i-5p | -2.077 | 1.166925985 |
| hsa-miR-192-5p | -6.0164 | 8.022648592 | hsa-miR-21-5p | -1.9123 | 1.14689121 |
| hsa-miR-381-3p | 1.0906 | 7.676603282 | hsa-miR-186-5p | -3.6315 | 1.068001035 |
| hsa-miR-7-5p | 2.9986 | 7.421135214 | hsa-miR-27a-3p | -2.4163 | 1.062696097 |
| hsa-miR-1246 | -0.1922 | 7.218738579 | hsa-let-7b-5p | -3.4934 | 0.948794384 |
| hsa-miR-26b-5p | -5.4961 | 7.062163573 | hsa-miR-100-5p | -3.9248 | 0.79982914 |
| hsa-miR-3529-3p | 0.5203 | 6.681407063 | hsa-miR-200a-5p | -4.1181 | 0.76570742 |
| hsa-let-7a-5p | -5.867 | 6.511175254 | hsa-miR-501-3p | -4.0769 | 0.743297024 |
| hsa-miR-146a-5p | 0.9996 | 6.479410504 | hsa-miR-92a-3p | -1.951 | 0.736891358 |
| hsa-let-7e-5p | -5.2753 | 5.957318491 | hsa-miR-99b-3p | -4.0366 | 0.70501178 |
| hsa-miR-30c-2-3p | -5.9021 | 5.523377122 | hsa-miR-98-5p | -3.3547 | 0.69599478 |
| hsa-miR-27b-3p | -5.617 | 5.31594998 | hsa-miR-328-3p | -2.141 | 0.653335034 |
| hsa-miR-429 | -5.9202 | 4.966668659 | hsa-miR-423-3p | -2.7508 | 0.615270163 |
| hsa-miR-30a-3p | -5.7227 | 4.782421094 | hsa-miR-200b-5p | -4.2191 | 0.568069821 |
| hsa-miR-26a-5p | -5.5616 | 4.493624363 | hsa-miR-103b | -3.9933 | 0.534750697 |
| hsa-miR-1-3p | 0.2605 | 4.462315765 | hsa-miR-320a | -3.0567 | 0.520431821 |
| hsa-miR-30e-3p | -5.6009 | 4.297993174 | hsa-miR-361-3p | -2.1498 | 0.51436643 |
| hsa-miR-375 | -5.7609 | 4.175848287 | hsa-miR-10a-5p | -4.2379 | 0.507378245 |
| hsa-miR-128-3p | -1.3263 | 4.008137576 | hsa-miR-151a-3p | -3.7918 | 0.491309376 |
| hsa-miR-146b-5p | -4.686 | 3.998595343 | hsa-miR-20a-5p | -2.6302 | 0.485390607 |
| hsa-miR-3184-3p | -0.1756 | 3.906640982 | hsa-miR-183-5p | -2.1258 | 0.462025859 |
| hsa-miR-200a-3p | -5.4709 | 3.899596565 | hsa-miR-125b-5p | -3.8403 | 0.446798836 |
| hsa-miR-23a-3p | -4.7124 | 3.856452164 | hsa-miR-24-3p | -3.9177 | 0.443493218 |
| hsa-miR-200c-3p | -5.5082 | 3.559475898 | hsa-miR-203a-3p | -3.3207 | 0.390395162 |
| hsa-miR-204-5p | -5.4601 | 3.46523836 | hsa-miR-744-5p | -3.543 | 0.379006198 |
| hsa-miR-155-5p | -0.1446 | 3.43116131 | hsa-miR-148b-3p | -3.3947 | 0.33277383 |
| hsa-let-7f-5p | -5.0875 | 3.351389635 | hsa-miR-25-3p | -2.8272 | 0.303236928 |
| hsa-miR-200b-3p | -5.1632 | 3.04890351 | hsa-miR-31-5p | -2.7243 | 0.270953728 |
| hsa-miR-7704 | -0.6161 | 3.014819449 | hsa-miR-17-5p | -2.1879 | 0.233044305 |
| hsa-miR-222-3p | -1.0987 | 2.991587798 | hsa-miR-210-3p | -2.5542 | 0.222069066 |
| hsa-miR-7641 | -0.7473 | 2.880724594 | hsa-miR-16-5p | -2.9124 | 0.205026232 |
| hsa-miR-30d-5p | -5.0678 | 2.719888696 | hsa-miR-93-5p | -2.4891 | 0.1837974 |
| hsa-miR-141-3p | -5.0392 | 2.677733102 | hsa-miR-30e-5p | -3.2304 | 0.154182845 |
| hsa-miR-27a-5p | -0.9607 | 2.663119672 | hsa-miR-664a-5p | -3 | 0.129508016 |
| hsa-miR-532-5p | -4.5412 | 2.648046462 | hsa-miR-99b-5p | -3.6798 | 0.128429671 |
| hsa-miR-221-3p | -1.1017 | 2.576359113 | hsa-miR-205-5p | -3.2881 | 0.124453708 |
| hsa-miR-129-5p | -0.901 | 2.490516584 | hsa-miR-3074-5p | -3.1716 | 0.102324892 |
| hsa-miR-148a-3p | -4.7132 | 2.277842894 | hsa-miR-584-5p | -3.4786 | 0.071825589 |
| hsa-miR-92b-3p | -0.9847 | 2.22668648 | hsa-miR-22-3p | -2.8189 | 0.05390251 |
| hsa-miR-125a-5p | -2.1037 | 2.198845919 | hsa-miR-185-5p | -2.5998 | 0.052230343 |
| hsa-miR-500a-3p | -4.7899 | 2.184434523 | hsa-miR-182-5p | -2.9196 | 0.04737366 |
| hsa-miR-455-5p | -4.3865 | 2.180351034 | hsa-miR-181b-5p | -2.6916 | 0.044390771 |
| hsa-miR-218-5p | -4.5802 | 2.133615735 | hsa-let-7d-3p | -2.99 | 0.04220461 |
| hsa-miR-941 | -0.9473 | 2.098298623 | hsa-miR-106b-3p | -2.6906 | 0.039249342 |
| hsa-miR-125b-2-3p | -5.046 | 2.07505742 | hsa-miR-181a-5p | -3.388 | 0 |
