# Supplementary material for: Urinary exosome miR‐30c‐5p as a biomarker of clear cell renal cell carcinoma that inhibits progression by targeting HSPA5
Source: J Cell Mol Med. 2019 Jul 24;23(10):6755–65. doi: 10.1111/jcmm.14553 (PMC6787446; doi:10.1111/jcmm.14553)
Supplement: Supplementary file 3 [file JCMM-23-6755-s003.docx]

Table S2 49 miRNAs having similar expression difference trend in urinary and cellular exosomes

| miRNA_ID | normal(n=3) | ccRCC(n=3) | HK-2(n=3) | 786-0(n=3) | ACHN(n=3) |
| --- | --- | --- | --- | --- | --- |
| hsa-miR-23b-3p | 119.4849411 | 1 | 4.638866245 | 2.157779449 | 1 |
| hsa-miR-194-5p | 76.69928707 | 1.34466754 | 4.257187868 | 0.807225895 | 1 |
| hsa-miR-30c-5p | 977.1973811 | 17.3465008 | 139.0485747 | 4.887643007 | 7.461527762 |
| hsa-miR-30a-5p | 3513.009748 | 65.56134148 | 3502.550192 | 112.6547841 | 47.99992236 |
| hsa-miR-30c-2-3p | 38.6745235 | 0.970114943 | 4.550787366 | 2.25092369 | 1 |
| hsa-miR-30a-3p | 408.8254329 | 11.61278918 | 181.7378951 | 166.0715035 | 59.84605929 |
| hsa-miR-27b-3p | 377.1860614 | 11.42677142 | 304.8146746 | 199.1057295 | 6.538449594 |
| hsa-miR-26a-5p | 1040.251244 | 32.73670886 | 191.573467 | 106.0572556 | 19.84612075 |
| hsa-miR-26b-5p | 128.6443765 | 4.275643824 | 30.18199669 | 21.60885357 | 1.538459299 |
| hsa-miR-200a-3p | 209.3673214 | 7.080896261 | 45.7427539 | 24.46519668 | 1.384612399 |
| hsa-let-7f-5p | 1506.71986 | 65.94891605 | 173.252879 | 123.0245537 | 21.84611752 |
| hsa-miR-30d-5p | 1179.504554 | 52.50154227 | 342.7183274 | 205.2065041 | 18.84612399 |
| hsa-miR-99a-5p | 836.4071002 | 43.86796159 | 6.312381119 | 49.42715055 | 3.615377897 |
| hsa-miR-500a-3p | 10.0091372 | 0.542717882 | 0.939517746 | 0.481232598 | 0.230768733 |
| hsa-miR-101-3p | 109.4258111 | 6.575585625 | 63.18254338 | 50.70008775 | 2.384612399 |
| hsa-miR-152-3p | 36.97509094 | 2.244594791 | 57.04632002 | 22.88179077 | 5.307684096 |
| hsa-let-7g-5p | 477.3649644 | 31.0883748 | 275.9245144 | 196.4356716 | 40.07686037 |
| hsa-miR-103b | 101.3785247 | 9.399927252 | 54.0222494 | 4.610509613 | 1.153843665 |
| hsa-miR-378a-3p | 251.9280372 | 23.58254038 | 220.9040248 | 170.3249706 | 67.84604635 |
| hsa-miR-140-3p | 55.06902372 | 5.487792812 | 58.92535551 | 2.654536638 | 5.076915363 |
| hsa-miR-151a-3p | 340.835734 | 36.64228139 | 303.1117969 | 1595.733228 | 128.076718 |
| hsa-miR-29a-3p | 26.46611378 | 3.394674814 | 176.8935083 | 75.94146444 | 12.15382749 |
| hsa-miR-584-5p | 1.886861632 | 0.253906591 | 3.435111862 | 0.946940607 | 0.230768733 |
| hsa-miR-148b-3p | 40.47394151 | 5.72604394 | 63.0944645 | 52.33006413 | 4.99999353 |
| hsa-miR-203a-3p | 60.47973229 | 9.079906882 | 1.556076369 | 0.031045881 | 0.076921833 |
| hsa-miR-30e-5p | 32.75152044 | 5.17951404 | 94.39213669 | 2.142258158 | 0.769228032 |
| hsa-miR-182-5p | 37.15003637 | 7.364906155 | 157.2517242 | 61.90812913 | 42.69223827 |
| hsa-miR-22-3p | 42.8606431 | 9.039473301 | 61.56774776 | 8.180937677 | 1.384612399 |
| hsa-miR-20a-5p | 16.15707842 | 3.859624618 | 150.7338224 | 19.28031353 | 11.07690566 |
| hsa-miR-185-5p | 8.384693729 | 2.074770842 | 18.29122834 | 7.994655794 | 2.307687331 |
| hsa-miR-210-3p | 2.536650662 | 0.647824822 | 14.26892072 | 2.188828629 | 0.230768733 |
| hsa-miR-93-5p | 10.17159901 | 2.71769242 | 102.6716354 | 8.957117691 | 2.230765498 |
| hsa-miR-27a-3p | 13.02063146 | 3.608104176 | 215.2082023 | 108.0908449 | 4.99999353 |
| hsa-miR-21-5p | 642.1347301 | 254.0978175 | 7657.419051 | 1559.392473 | 278.3072474 |
| hsa-miR-193a-5p | 1.536970755 | 0.6744071 | 1.82031624 | 2.141512608 | 1.923074932 |
| hsa-miR-615-3p | 1.012163538 | 0.499854503 | 0.440397629 | 0.873728277 | 1.153843665 |
| hsa-miR-9-5p | 21.94264513 | 11.30715845 | 4.374626373 | 5.01412256 | 3.619479704 |
| hsa-miR-361-3p | 6.472835734 | 3.642848829 | 4.785667611 | 6.318105644 | 5.769221562 |
| hsa-miR-27a-5p | 0.999650808 | 0.770435036 | 1.7028745 | 0.807225895 | 2.230765498 |
| hsa-miR-340-5p | 6.272893933 | 5.572632038 | 18.64354709 | 18.75251376 | 13.40099375 |
| hsa-miR-7641 | 0.56231631 | 0.502487997 | 9.718133354 | 15.39941346 | 12.96390862 |
| hsa-miR-423-3p | 23.35466317 | 22.58806926 | 46.21251116 | 46.12062402 | 32.15379514 |
| hsa-miR-184 | 1.911857995 | 2.544361996 | 1.790956614 | 2.142258158 | 2.846149865 |
| hsa-miR-486-5p | 1.624443474 | 4.137130802 | 1.673514874 | 4.424227729 | 8.230755794 |
| hsa-miR-381-3p | 0.874698094 | 4.03583588 | 2.172634991 | 0.512278479 | 16.46151159 |
| hsa-miR-451a | 2.998981522 | 14.3884039 | 1.379915376 | 3.104720056 | 5.923068463 |
| hsa-miR-3591-3p | 1.012163538 | 6.233158737 | 0.822079241 | 8.460363802 | 11.99998059 |
| hsa-miR-122-5p | 1.287065328 | 9.122668413 | 3.904869117 | 16.47054419 | 31.92302641 |
| hsa-miR-4497 | 0.46233086 | 3.330306998 | 7.105100734 | 30.59702044 | 23.02046375 |
